# Mesenchymal Stem Cell Expressing TRAIL as Targeted Therapy against Sensitised Tumour

**DOI:** 10.3390/ijms19082188

**Published:** 2018-07-27

**Authors:** Kamal Shaik Fakiruddin, Nadiah Ghazalli, Moon Nian Lim, Zubaidah Zakaria, Syahril Abdullah

**Affiliations:** 1 Stem Cell Laboratory, Haematology Unit, Cancer Research Centre, Institute for Medical Research, Kuala Lumpur 50588, Malaysia; limmn@imr.gov.my (M.N.L.); zubaidah@imr.gov.my (Z.Z.); 2 UPM-MAKNA Cancer Research Laboratory, Institute of Bioscience, Universiti Putra Malaysia, Serdang 43400, Selangor, Malaysia; syahril@upm.edu.my; 3 Medical Genetics Laboratory, Department of Biomedical Sciences, Faculty of Medicine & Health Sciences, Universiti Putra Malaysia, Serdang 43400, Selangor, Malaysia; nadiahwmg@upm.edu.my

**Keywords:** mesenchymal stem cells, TRAIL, apoptosis, sensitisation, cancer stem cells, tumours

## Abstract

Tapping into the ability of engineered mesenchymal stem cells (MSCs) to mobilise into the tumour has expanded the scope of cancer treatment. Engineered MSCs expressing tumour necrosis factor (TNF)-related apoptosis inducing ligand (MSC-TRAIL) could serve as a platform for an efficient and targeted form of therapy. However, the presence of cancer stem cells (CSCs) that are resistant to TRAIL and apoptosis may represent a challenge for effective treatment. Nonetheless, with the discovery of small molecular inhibitors that could target CSCs and tumour signalling pathways, a higher efficacy of MSC-TRAIL mediated tumour inhibition can be achieved. This might pave the way for a more effective form of combined therapy, which leads to a better treatment outcome. In this review, we first discuss the tumour-homing capacity of MSCs, its effect in tumour tropism, the different approach behind genetically-engineered MSCs, and the efficacy and safety of each agent delivered by these MSCs. Then, we focus on how sensitisation of CSCs and tumours using small molecular inhibitors can increase the effect of these cells to either TRAIL or MSC-TRAIL mediated inhibition. In the conclusion, we address a few questions and safety concerns regarding the utilization of engineered MSCs for future treatment in patients.

## 1. Introduction

The GLOBOCAN 2012 report published by the World Health Organization estimates that there were about 14.1 million new cancer cases, 8.2 million cancer deaths, and 32.6 million people living with cancer in 2012 [1]. It was predicted that in 2025, there would be a sharp increase in new cancer cases, of up to 19.3 million total cases, because of the ageing population [1]. Despite considerable advances in our knowledge and experience in the treatment of cancer, our capacity to effectively fight and treat the disease is still limited [2]. Current treatments only manage to reduce the burden of the primary lesion but are rarely effective in the complete eradication of tumour cells, which in turn leads to relapse and even fatality [3]. This is due to the existence of chemotherapy-resistant cancer stem cells (CSCs) that can repopulate the tumour after the initial chemotherapy [4]. This warrants the need for a more efficient and innovative approach that can enhance treatment efficacy in cancer patients. The idea of using mesenchymal stem cells (MSCs) as vectors for anti-tumour ligand delivery, such as tumour necrosis factor (TNF)-related apoptosis inducing ligand (TRAIL), has emerged as one of the avenues of cytotherapy in cancer treatment, as these cells were shown to home the tumour site and deliver targeted therapies. Furthermore, with the use of small molecular inhibitors in CSCs and tumours to enhance the sensitivity of these cells to TRAIL or MSC-TRAIL mediated inhibition, better treatment efficacy can be achieved. This review will first look into the characteristics of MSCs, its effect on tumour tropism, the tumour-directed homing of MSCs, and the anti-cancer properties of engineered MSCs that have been reported. The review will further focus on TRAIL in the treatment of cancers, the idea of cancer stem cells, resistance of tumour and CSCs to TRAIL, sensitisation of CSCs, and tumour to TRAIL-mediated inhibition, and the use of MSCs expressing TRAIL or MSC-TRAIL to target sensitised CSCs and tumours.

## 2. Mesenchymal Stem Cells

The multipotent characteristic of human mesenchymal stem cells (MSCs) is an exclusive feature, which is not seen in any other mature cells [5]. MSCs can be isolated from various sources, such as bone marrow [6], umbilical cord blood [7], and adipose tissue [8], and can be cultured and stably expanded for several passages while retaining its characteristics [9]. Compared to other potential cytotherapy, MSCs are relatively non-immunogenic, thus overcoming the difficulties of immune rejection caused by transplanted cells [10]. These characteristics make MSCs an attractive candidate for cell-based therapy for degenerative diseases [11]. MSCs also express specific surface markers, such as (cluster of differentiation) CD73, CD90, and CD105, while lacking other markers, such as CD34, CD45, major histocompatibility complex (MHC) II, and hematopoietic stem cell markers [12]. Another unique characteristic of MSCs compared to other adult stem cells, lies in the capacity of these cells to avoid an immune response, because of the lack of MHC II and its co-stimulatory molecules (CD86 and CD40), thereby reducing the risk of graft versus host rejection [13,14,15]. Accordingly, MSCs are great candidates for bio-banking and autologous transplants [16]. These cells are also malleable to genetic engineering, and have been shown to have the capacity to robustly express exogenous proteins [17]. These qualities have paved the way to use MSCs not just for the treatment of degenerative diseases, but as cytotherapeutic-based vector for the treatment of various tumours.

## 3. MSCs and Its Effects in Tumour Tropism

The enhancement of the proliferative, resistance, and aggressive phenotypes of tumour cells has been the subject of intense investigation. Most studies propose that the phenotypes are solely acquired through genomic instability and abnormal cellular changes within the tumour cells [18], while others view these characteristics as a process activated through the paracrine factors released by the tumour microenvironment (TME) [19,20,21]. It has been shown that MSCs secrete microvesicles and exosomes containing an array of cytokines, chemokines, and growth factors that regulate cellular growth, angiogenesis, and inflammation [22,23]. As MSCs are also part of the stromal cells that reside within the TME, it is expected that MSCs may contribute either to the development or inhibition of a tumour. MSCs are also known to affect the proliferation and differentiation of dendritic cells, macrophages, B and T cells, natural killer cells (NK cells), and mast cells [24].

**Figure 1:**
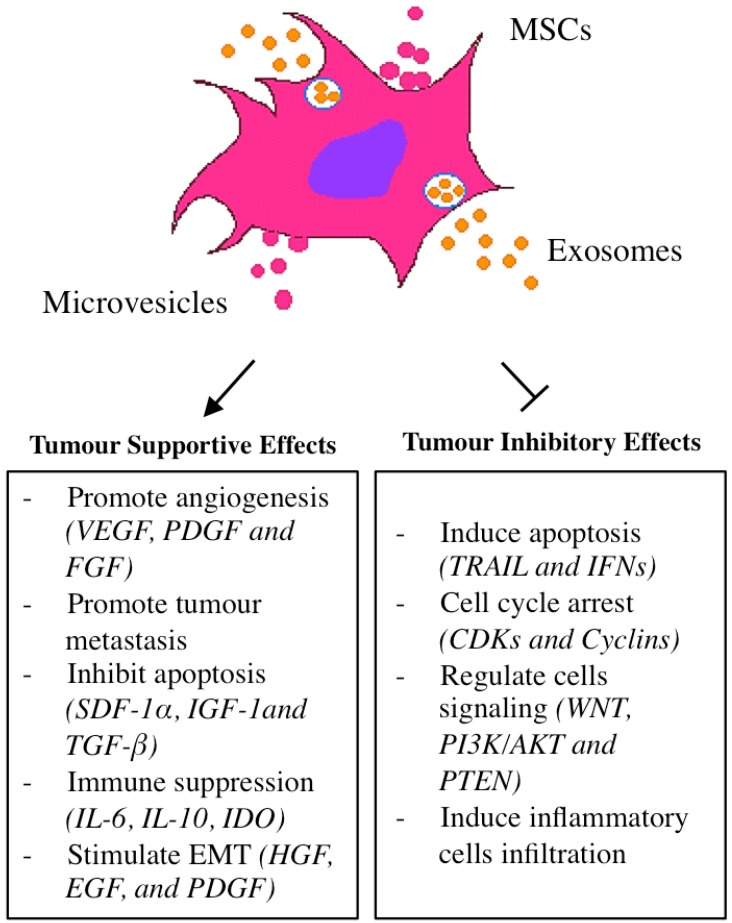
Engineered mesenchymal stem cells (MSCs) act to support and inhibit tumour growth. MSCs could induce apoptosis in some tumours, while others have reported that MSCs might also inhibit apoptosis. MSCs could promote vascularization in the tumour microenvironment by secreting growth factors and might also lead to tumour inhibition by inducing cyclin dependent kinases (CDKs) and cyclins block that leads to cell cycle arrest. These ambiguous roles of MSCs suggested that more studies are needed to elucidate the exact function of MSCs in different tumour models for a safer treatment outcome. TRAIL—tumour necrosis factor-related apoptosis inducing ligand; VEGF—vascular endothelial growth factor; PDGF—platelet-derived growth factor; FGF—fibroblast growth factors; IFN—interferon; IGF—insulin-like growth factor; TGF—transforming growth factor; IDO—indoleamine 2,3-dioxygenase; HGF—hepatocyte growth factor; EGF—epidermal growth factor; PDGF—platelet-derived growth factor; WNT—proto-oncogene protein; IL—interleukin; SDF—stromal derived factor one alpha; AKT—serine/threonine kinase; PTEN—phosphatase and tensin homolog.

Although a number of studies have shown that native MSCs are capable of inducing tumour suppression and apoptosis, as seen in hepatoma [25], leukemia [26], and Kaposi’s Sarcoma [27], others have demonstrated an opposite effect [28,29,30]. A recent study has proposed that therapy-educated MSCs can enhance the resistant characteristic of pancreatic adenocarcinoma to therapy by enriching CSCs [31]. The ambiguous role of MSCs during tumour development is attributed to the heterogenic characteristics of MSCs that are the product of the MSC origin and growth conditions [32,33]. Indeed, it is difficult to define the complex role of MSCs, as most studies were performed using MSCs from different sources with varying conditions [34]. Despite the numerous studies that have advanced our understanding of the biology of MSCs, leading to their subsequent applications in cancer therapy, more studies are needed to fully understand MSCs’ influence on various tumour types. Figure 1 summarizes the inhibitory and supportive effects of MSCs and the molecules that play a role in the process.

## 4. Tumour Homing Capacity of Mesenchymal Stem Cells (MSCs)

The ability of the transplanted MSCs to home the tumour microenvironment has expanded the therapeutic benefits of these cells beyond their use in degenerative diseases [35]. Numerous reports have shown that MSCs are capable of infiltrating into the tumour stroma and its microenvironment, and possibly contributing towards stromal support [36]. However, the definitive role of MSCs within the tumour stroma is unknown. The exact mechanism in which MSCs migrate into the tumour microenvironment is not fully understood. However, it is widely accepted that the secretion of chemokines and cytokines from the tumour microenvironment and the expression of conjugate receptors on MSCs are possible causes. Although the identities of the cytokines and chemokines, as well as their respective receptors, are not yet known, it is postulated that a combination of several receptors and ligands contributes to the homing characteristic.

One specific ligand, chemokine (C-X-C motif) ligand 12 (CXCL12), secreted by the tumours, with its concomitant receptor (C-X-C chemokine) receptor type 4 (CXCR4), which is expressed mostly in MSCs, has drawn particular interest, given its connection to the tumour-homing characteristic of MSCs as well as its contribution to MSCs’ migration [37,38]. Some studies have suggested that both CXCL12 and CXCR4 contributed significantly during angiogenesis and hematopoietic stem cell mobilization, while others have suggested its major contribution during tumour development [39,40]. Although several studies have shown a strong connection between CXCL12/CXCR4 signalling towards MSCs migration and the tumour homing capacity, the knockdown of these receptors does not inhibit the homing potential of MSCs [41]. This may be due to the fact that some MSCs do not express the receptor at all, and CXCL12/CXCR4 may not be the only molecules that influence the MSC migration. Several studies have also suggested the ability of MSCs to home in on injured and inflamed tissues, such as in cases of acute lung injury [42] and the liver cirrhotic model [43], indicating MSCs’ paracrine and direct effects on regulating and healing of the damaged tissue.

## 5. Engineered MSCs for Anti-Tumour Therapy

### 5.1. Delivery of Anti-Tumour Cytokines

MSCs derived from the bone marrow, adipose tissue, and umbilical cord have been used as a delivery vehicle for targeted anti-tumour therapies [44]. These immunoprivileged cells, in addition to their reduced rejection risk, can home in on the tumour microenvironment, thus enhancing their potential for use in allogeneic transplantation and cytotherapeutics [45,46,47,48,49]. Several studies that altered the genes of these cells demonstrated that the exogenous expression of therapeutic genes, such as bone morphogenic protein 2 (BMP-2), B-cell lymphoma 2 (BCL-2), and erythropoietin (EPO) has enhanced the treatment efficacy of MSCs at the target site [50,51,52]. This leads to the idea of using genetically-engineered MSCs as a vehicle to deliver biological anti-tumour agents directly at the tumour microenvironment [45,53]. Many studies have shown that MSCs engineered to express anti-tumour cytokines, such as interleukin-2 (IL-2) [54], interferon-beta (IFN-β) [55], TRAIL [45,46,48], and IL-15 [56], are able to deliver these ligands directly to the tumour site and to efficiently induce tumour regression. Moreover, the use of non-viral gene-delivery techniques has also been studied in MSCs, suggesting an effective and yet safer method for gene-delivery to MSCs [57]. A previous work using transfected MSCs derived from adipose tissue has shown that when these cells express a potent anti-tumour agent called TRAIL, the engineered adult stem cells (termed MSC-TRAIL) are capable of inducing apoptosis in glioblastoma (LN18) and hepatocellular carcinoma (HepG2) cells in vitro [58].

### 5.2. Delivery of Pro-Drug Converting Enzymes

Preclinical studies have shown that engineered MSCs expressing pro-drug converting enzymes are useful for the treatment of late stage tumours and for the prevention of metastasis [59]. With this strategy, the off-site accumulation of the active drug can be prevented, thus reducing the treatment toxicity. An example of a pro-drug converting system is the yeast cytosine deaminase/5-fluorocytosine (5-FC), which uses MSCs to locally deliver yeast cytosine deaminase (yCD) to the tumour site. The conversion of 5-FC to 5-fluorouracil (5-FU) by yCD induces cytotoxic tumour regression in several cancers [60,61]. Another example of an MSC-mediated pro-drug converting system is the thymidine kinase/ganciclovir system and nitroreductase/CB1954 system. Both systems have been extensively studied in several tumour models, with promising effects [62,63].

### 5.3. MSCs as Vectors for Oncolytic Viruses

In addition to the utilization of MSCs as vectors to deliver cytokines and pro-drug enzymes into tumours, MSCs have also been studied as a vehicle to deliver oncolytic viruses to the tumour. Oncolytic viruses are viruses that induce tumour regression by direct tumour cell oncolysis [64,65] and the disruption of the tumour microenvironment [66]. Several reports have shown that the delivery of these viruses by MSCs enhanced the oncolytic effects of the virus in several tumour models [67,68,69,70]. The delivery of these viruses led to the destruction of tumour cells, as the viruses replicate and spread to the surrounding stroma, which further induces tumour regression. Among the viruses that have been studied, three types of viruses, namely the adenovirus, the measles virus, and the herpes simplex virus have been shown to have a highly significant impact on reducing tumour growth [71]. The MSCs loaded with oncolytic viruses were effective in reducing tumour metastasis in several models, as seen in lung cancer [72], glioma [73], and breast cancer [74]. The direct inhibition of tumour growth was also observed in hepatocellular carcinoma, pancreatic cancer, brain tumour, and non-small cell lung cancer (NSCLC), in both in vitro and in vivo studies.

### 5.4. Safety Profile of Engineered MSCs

A different approach to using engineered MSCs has highlighted different anti-tumour efficacies and several safety concerns. As a result of the expression of the TRAIL receptors (DR4 and DR5), which are highly expressed in tumours, compared to normal cells, the efficacy of TRAIL to induce tumour regression is higher, and the toxicity effect of TRAIL on normal cells is lower, compared with other cytokines [75]. Engineered MSCs expressing pro-drug converting enzymes may enhance the effects of chemotherapy by localized drug activation. However, if factors such as the number of migrated MSCs to the tumour and the level of enzymes at the target site are not fully optimised, these factors may hamper the overall treatment outcome in patients [76]. Although oncolytic viruses have emerged recently as a potential agent in cancer treatment, the efficacy and safety of using this approach for cancer treatment have been hindered because of the low virus spread at the tumour surrounding [77] and the probability of these viruses reverting to their wild type, thus infecting the normal cells [78]. This approach of using MSCs as a vehicle for the delivery of anti-tumour agents and its safety are summarized in Table 1.

## 6. Tumour Necrosis Factor-Related Apoptosis Inducing Ligand (TRAIL) and Cancer Treatment

### 6.1. Tumour Necrosis Factor (TNF)-Related Apoptosis Inducing Ligand (TRAIL)

The tumour necrosis factor related apoptosis inducing ligand (TRAIL), also known as Apo2L, is one of several members of the TNF gene superfamily that induces apoptosis. Its mechanism of action is by activating the extrinsic apoptosis pathways through binding its two specific agonistic receptors (TRAIL-R1/DR4 and TRAIL-R2/DR5) and three antagonistic decoy receptors [TRAIL-R3/DcR1, TRAIL-R4/DcR2, and osteoprotegerin (OPG)] [90]. The TRAIL protein can either be a soluble ligand or attached as a transmembrane protein by a hydrophobic amino acid bond. TRAIL is expressed in a variety of normal tissues, such as the placenta, kidney, and spleen, and is secreted into the peripheral blood because of the inflammatory response [91], viral infections [92], and malignant diseases [93]. Several studies have documented the efficacy of TRAIL as a potent anti tumour agent on its own [94,95,96], while others have recommended TRAIL as a combination treatment because of the possible resistance in some tumour models [97,98,99]. These studies are detailed and elaborated in the next sections.

### 6.2. TRAIL Treatment in Solid Tumours

Several studies have documented the efficacy of TRAIL in inhibiting the proliferation and inducing apoptosis in vitro, in a variety of tumours, including colorectal cancer [100], glioblastoma [101], and NSCLC [102]. Furthermore, TRAIL has also been reported to inhibit the proliferation of several chemoresistant cancer cell lines [103,104]. In small animal models, TRAIL-induced tumour regression was well documented in colon and breast carcinoma of SCID mice [105,106]. However, some have described the poor bioavailability and short half-life of TRAIL upon administration to a xenograft model, which eventually resulted in poor bioavailability of the ligand [105]. Nonetheless, modifications of the TRAIL protein structure and fusion with other immunoglobulin molecules have significantly prolonged its half-life and perhaps even enhanced its anti-tumour activity [107].

### 6.3. Synergistic Effects of TRAIL-Based Combination Therapy

The pre-treatment of tumour cells by small molecule inhibitors have been shown to increase the sensitivity of TRAIL-induced apoptosis [108]. These molecules include the inhibitors of mammalian target of rapamycin (mTOR) [109], proteasome [110], histone deacetylases (HDAC) [111], and BCL-2 [112], and they have been used in combination with recombinant TRAIL to inhibit specific signalling molecules that would interfere with the extrinsic activation of apoptosis by TRAIL. In lung cancer, compounds such as bortezomib [102], cardiac glycosides [113], and transhinone IIA [114] have been reported to have synergistic or sensitising effects on TRAIL-mediated apoptosis. The pre-treatment of tumour cells by standard chemotherapy drugs have also been shown to be a promising approach, based on several in vitro studies [97,99,115]. However, all of these approaches, which often target non-CSCs, may not be able to eradicate the tumour completely. Strategies to target TRAIL-resistant CSC populations should be explored for better treatment efficacy.

## 7. The Existence of Cancer Stem Cells

Tumours are composed of heterogeneous populations of cells. Each sub-population varies in its differentiation, proliferation, and tumourigenic capacity [116,117]. In vivo models have demonstrated that a small sub-population of cells has strong stem cell or pluripotent characteristics [118]. They are known as cancer stem cells (CSCs), or cancer initiating cells, and are able to initiate tumour development in vivo [119]. Classical chemotherapy may reduce the bulk of the tumour and improve the patient’s quality of life, but because of the strong chemoresistant characteristic of CSCs from high aldehyde dehydrogenase (ALDH) enzyme activity, enhanced DNA repair mechanism, and the efflux of drugs by the adenosine triphosphate (ATP)-binding cassette or ATP-binding cassette transporters (ABC) transporters, most patients that underwent chemotherapy eventually experienced relapse. Therefore, it is by identifying and therapeutically targeting these stem cells that the response and outcome of treatments could be improved.

The cluster of differentiation (CD) molecules have been used as the most reliable technique for the isolation and identification of cell populations enriched with stem cell properties. One such example is CD133, which has recently been identified as the marker for CSC in lung cancer [120], prostate cancer [121,122,123], brain cancer [124,125,126,127], colon cancer [128,129,130], and hepatic carcinoma [131,132,133,134]. CSCs are also identified as the side population (SP), based on the expression of the ABCG2 protein and the ability to efflux Hoechst dye [135]. In a recent study, Lim and his group identified a combination of CD166-positive and Lin-negative sub-population of lung cancer cells that link a glycine metabolism enzyme to tumour formation as a novel therapy targeting a specific metabolic pathway in NSCLC [136]. In the identification of the CSCs derived from non-small cell lung cancer, markers such as the CD133, SP population, and ALDH 1 have been extensively studied and reported [137,138,139]. We have recently identified and characterised a novel double positive (CD166^+^/CD44^+^ and CD166^+^/EpCAM^+^) CSC sub-population isolated from NSCLC cell lines (A549 and H2170), and showed that these two sub populations exhibit a self-renewal capacity, higher mobility, resistance to apoptosis, and the ability to differentiate towards the mesenchymal lineage [140]. A list of CSC markers and the type of tumours are summarized in Table 2.

## 8. Resistance of CSCs to TRAIL and Apoptosis

CSCs are known for being highly resistant to apoptosis even through the stimulation of the TRAIL death ligand. In general, the remarkably impaired regulation of apoptosis in CSCs compared to non-CSCs are because of the lower expression of death signals (i.e., CASP8/caspase 8, CASP3/caspase 3, and PARP/Poly [adenosine diphosphate ribose (ADP-ribose)] polymerase 1) and the higher expression of anti-apoptotic molecules (i.e., cFLIP/cellular FLICE-like inhibitory protein, BCL-2/B-cell lymphoma 2, and XIAP/inhibitors of apoptosis), leading to the characteristics of CSCs’ being highly resistant to apoptosis [151]. Other factors include the tumour microenvironment, genetics, epigenetics, and inter- and intra-tumour heterogeneity, which may also contribute to the resistance. High expressions of the DR4 and DR5 receptors, which are the agonistic TRAIL receptors, were reported as the contributor of CSCs’ resistance to TRAIL-mediated effects and the chemo-resistant characteristic observed in colon cancer [152]. However, in glioblastoma, the low expression of the death receptor (DR4 and DR5) and high expression of cFLIP, a master anti-apoptotic regulator molecule, led to the glioblastoma-derived CSCs resistance to TRAIL [153]. The activation of the extrinsic apoptotic pathways through DR4 and DR5 ligand-activation promotes the expression of various apoptosis inhibitory proteins in CSCs that include the NF-κB, which also makes CSCs resistant to TRAIL-based therapy, as seen in glioblastoma [154]. Other anti-apoptotic signalling molecules, such as X-linked inhibitor of apoptosis proteins (XIAPs), were also observed to reduce the effects of TRAIL-mediated apoptosis in CSCs derived from nasopharyngeal carcinoma [155].

## 9. Sensitisation of CSCs to TRAIL and Apoptosis

The ability of CSCs to evade apoptotic signals contributes towards chemoresistance in most cancers. Therefore, therapeutic strategies that can enhance the onset of apoptosis in CSCs may serve as a more promising approach. It is known that the high expression of anti-apoptotic genes in CSCs makes these cells highly resistant to cell death and apoptosis, which contributes greatly to cancer progression [136,156,157]. These anti-apoptotic genes present potential therapeutic targets, particularly to discriminate CSCs from non-CSCs [158]. It has been shown that, by regulating specific anti-apoptotic genes through gene knock down or silencing, the sensitivity of CSCs toward therapy can be enhanced [4]. This means that combination therapies can sensitize CSCs against TRAIL, and common chemotherapy might be an ideal approach for effective treatment.

Chemoresistance of tumour cells that contributes towards cancer recurrence is mostly comprised of a pool of CSCs that are TRAIL-resistant. Owing to the high cFLIP expression in most tumours, it is believed that the overexpression of cFLIP is also the main contributor to TRAIL-resistance in CSCs [159,160,161,162]. One study demonstrated that by regulating this molecule, the sensitivity of tumours to TRAIL-mediated apoptosis and to common chemotherapies, such as taxol, gemcitabine, and cisplatin, is enhanced [153,163,164]. In breast cancer stem cells, the silencing of cFLIP by siRNA or a chemical known as droxinostat [165], sensitised them to TRAIL-mediated effects, and the combination of both the cFLIP inhibition and the TRAIL induction resulted in a significant reduction in the tumour bulk, metastasis, and self-renewal of the breast CSCs. It was also shown that CD133-positive brain cancer stem cells expressed a high level of BCL-2 upon TRAIL induction, and a knockdown of BCL-2 subsequently enhanced the sensitivity of the CSCs to TRAIL-mediated inhibition [166]. Moreover, using second mitochondria-derived activator of caspases (SMAC) mimetics the induced inhibitor of apoptosis (IAP) degradation in nasopharyngeal carcinoma, and the effects of the TRAIL-mediated apoptosis was enhanced [155]. Finally, in colon cancer, the knockdown of Sirtuin 1 (SIR1) sensitised the colon CSCs to TRAIL-induced cytotoxicity [167].

## 10. Enhancing the Effect of MSC-TRAIL by Tumour Sensitisation

Mesenchymal stem cells, with their unique ability to home in on the tumour microenvironment and express exogenous transgenes, have garnered considerable interest as a viable therapeutic strategy. Engineered MSCs expressing TRAIL were able to kill the side population cells in the squamous and adenocarcinoma of the lung cancer cell lines, indicating the feasibility of these engineered cells to selectively kill putative cancer stem cells [168]. Similarly, the MSC-TRAIL was observed to significantly induce tumour cell death through caspase-mediated apoptosis in primary glioma-derived CD133 cells in vitro [169]. Moreover, the expression of TRAIL by MSCs enhanced the oncolytic effect of Newcastle disease virus (NDV) in glioma stem cells, resulting in positive synergistic effects compared to TRAIL or NDV alone [170]. In addition, the combination of MK886 (a lipoxygenase inhibitor) and MSC-TRAIL was beneficial in inducing the apoptosis of malignant glioma tumour cells via the upregulation of DR5, downregulation of anti-apoptotic protein survivin, and significant increase in the caspases’ activity [171]. All of these studies demonstrate that the MSC-TRAIL can selectively target CSCs, and further investigations to refine the approach for clinical applications are warranted.

Several reports have also suggested that, through the regulation of specific cellular signalling and proteins, the efficacy of MSCs expressing TRAIL to inhibit metastasis in several cancers was enhanced. For example, MSC-TRAIL has been shown to inhibit metastasis of the NSCLC derived-H460 cell line combined with Claudin-7, a small molecule that regulates mitogen-activated protein kinases/extracellular signal-regulated kinases (MEK/ERK) signalling pathways [172]. In pancreatic cancer, targeting of the XIAP [173] molecule resulted in the increased sensitivity to the MSC-TRAIL treatment and the suppression of metastasis [174]. It was shown in a metastatic renal cell carcinoma model that overexpressing thymidine kinase increased the sensitivity of the tumour cells to dodecameric TRAIL secreted by MSCs, and suggested that the combined administration of MSC-TRAIL and thymidine kinase is a potent strategy for the long-term remission of metastatic renal cell carcinoma [175].

**Figure 2:**
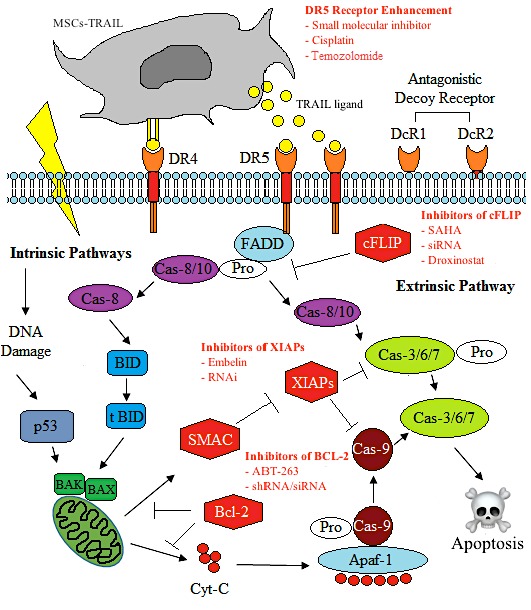
Sensitisation of tumour or cancer stem cells (CSC) to MSC-TRAIL induced apoptosis. An increase in the anti-apoptotic molecules (e.g., X-linked inhibitor of apoptosis proteins [XIAPs], cellular FLICE-like inhibitory protein [cFLIP], and B-cell lymphoma 2 [BCL-2]) upon TRAIL activation can be circumvented using specific inhibitors, as illustrated above. Through tumour sensitisation by anti-apoptosis gene silencing or specific DR5 receptor enhancement, and the utilization of MSCs as a vehicle for TRAIL delivery, a better treatment outcome could be achieved. SMAC—second mitochondria-derived activator of caspases; FADD—fas-associated protein with death domain; Cas—caspase; BID—BH3 interacting-domain death agonist; BAK—BCL-2 antagonist killer 1; BAX—BCL-2 associated X. Inhibitors: SAHA—suberoylanilide hydroxamic acid; shRNA—short hairpin RNA; siRNA—small interfering RNA; RNAi—RNA interference. The arrow and t-bar represent activated and inhibitory interactions respectively.

The dual effects of common chemotherapies, either as a cytotoxic drug or sensitiser to MSC-TRAIL, were recently described [176]. This was seen as a promising approach, especially to patients that have exhausted all available treatments. Recently, low dose cisplatin was able to increase the expression of TRAIL agonistic receptor DR4/5, and enhanced the efficacy of MSC-TRAIL, eventually decreasing tumour growth in glioblastoma multiforme [177] and hepatocellular carcinoma animal models [178]. A similar result was observed in a study using a mouse xenograft model of malignant glioma, where the administration of temozolomide enhanced the tumour sensitivity to MSC-TRAIL, by increasing the DR5 receptor expression and lowering the XIAPs and cFLIP expression [179]. Moreover, the sensitisation of human breast cancer cells by doxorubicin enhanced the apoptotic effect of the MSC-TRAIL and synergistically reduced the tumour growth in the xenograft mouse model [180]. A simplified diagram of the approach and the signalling involved upon TRAIL activation by MSC-delivery is illustrated in Figure 2.

### Challenges in MSC-TRAIL Applications: Discrepancies from In Vitro to In Vivo Models

The level of the TRAIL receptor expression does not correlate directly with the sensitivity of the tumour to the TRAIL-induced apoptosis [181,182]. Moreover, the sensitisation of these resistant tumour cells may yield a different effect towards TRAIL and MSC-TRAIL treatment in an in vitro and in vivo model. For example, in TRAIL-resistant colorectal carcinoma cells (CRC), subapoptotic genotoxic damage caused by 5-fluorouracil (5-FU) sensitised TRAIL-resistant CRC cells to MSC-TRAIL mediated inhibition in vitro. However, the sensitising effect was not achieved in an in vivo CRC mouse model. Rather, MSC-TRAIL seemed to support growth of the tumour, which invoked a cautionary warning, should the MSC-TRAIL be used in the clinic [183]. This may be due to the low intratumoural activity of 5-FU and sub-optimal tumour integration of MSC-TRAIL, which may hamper the overall treatment outcome [184]. It is also suggested that choosing the right tumour model that allows long term integration of the MSC-TRAIL to the target site is crucial for an effective in vivo model, as shown in the TRAIL-resistant medulloblastoma model [185]. It is expected that, by targeting the specific molecules that contributed to the TRAIL-resistant characteristics in these cells [186] and choosing a xenograft models with the most effective MSC-TRAIL integration, such as a pulmonary disease [187] or a metastatic model [188], a better treatment efficacy and tumour homing of MSC-TRAIL can be achieved.

## 11. Conclusions

Through an integrated approach, significant improvements have been made in the treatment of cancer. It was shown that the existence of cancer stem cell populations contributes to the challenges of developing an effective treatment in cancer. The regulation of specific molecules that lead to chemotherapy resistance characteristics in tumour cells and CSCs’ may represent as an ideal approach for a better treatment efficacy. Moreover, combining the tumour-homing capacity of MSCs and genetic engineering of the cells to express TRAIL-ligand, will enable the specific targeting of CSCs, thus paving the way towards a more effective treatment. However, several questions remain, such as the exact mechanism of the MSCs’ tumour-homing capacity and the fate of the MSCs after transfusion. These questions need to be answered to ensure the safety and efficacy of the treatment in future.

## Figures and Tables

**Table 1:** Biological agents utilizing engineered mesenchymal stem cells (MSCs) as vehicle for ligand delivery and its safety. TRAIL—tumour necrosis factor-related apoptosis inducing ligand; CSCs—cancer stem cells; IFN— interferon; IL—interleukin.

Biological Agents	Mechanism	Tumour Model	Toxicity and Safety Concern	References
IL-2	Reduce and inhibit tumour growth dependent of natural killer (NK) cells	Renal cell carcinoma, glioma	May cause capillary leak syndrome and fluid accumulation	[53,54,79]
IL-12	Inhibit tumour growth dependent of NK cells	Melanoma model, renal cell carcinoma	Haematological toxicity, such as neutropenia and thrombocytopenia	[80,81,82]
IL-15	Abolished tumour growth dependent of NK and CD8^+^ T cells	Pancreatic tumour	Probability for autoimmune toxicity	[56,83]
IL-18	Suppress proliferation, migration, and invasion	Breast tumour	Haematological toxicity, hypotension, and bradycardia	[84,85]
IFN-β	Inhibit tumour growth and metastasis in vivo	Melanoma, breast tumour	Haematological-, autoimmune-, and hepato-toxicity	[44,55,86]
TRAIL	Induce apoptosis, inhibit clonogenicity and tumour bulk	Lung metastasis, lung CSCs, glioma, pancreatic cancer, mesothelioma,	Mild constitutional toxicity (e.g., nausea, fever, and constipation) and anaemia	[45,47,48,58,87]
Pro-drug converting enzymes	Inhibition of tumour growth in vitro and in vivo	Glioma, prostate cancer, osteosarcoma	“Off site” activated drug accumulation	[59,60,61,63]
Oncolytic virus	Oncolytic viruses mediated tumour regression in vivo	Glioblastoma, brain metastasis, leukemia and pancreatic cancer	Potential for virus mutation, normal cell toxicity, and human viral transmission	[71,78,88,89]

**Table 2:** CSCs markers from different tumour types. ALDH—aldehyde dehydrogenase; SP—side population; ABCG2—ATP-binding cassette sub-family G member 2; CD—cluster of differentiation.

Cancer Type	CSCs Markers	References
Non-small cell lung cancer (NSCLC)	ABCG2^+^, CD133^+^, CD44^+^, EpCAM+, CD166^+^, ALDH^+^	[137,138,140]
Breast	CD44^+^/CD24^−^, ALDH^+^	[141,142]
Colon	CD133^+^, EpCAM high/CD44^+^	[128,129,143,144]
Head and neck	CD44^+^, SP, ALDH	[145,146]
Prostate	CD133^+^, CD44^+^, α2β1high	[147,148]
Brain tumour/glioma	CD133^+^, CD15^+^, CD90^+^, CD49f^+^	[126,149,150]

## Abbreviations

MSCs	mesenchymal stem cells
TRAIL	tumour necrosis factor related apoptosis inducing ligand
CSCs	cancer stem cells
IL	interleukin
IFN	interferon
NSCLC	non-small cell lung cancer
mTOR	mammalian target of rapamycin
HDAC	histone deacetylases
BCL-2	B-cell lymphoma 2
ABCG2	ATP-binding cassette sub-family G member 2
ALDH1	aldehyde dehydrogenase 1
PARP	poly (ADP-ribose) polymerase
cFLIP	cellular FLICE (FADD-like IL-1β-converting enzyme)-inhibitory protein
XIAP	X-linked inhibitor of apoptosis protein
SAHA	suberoylanilide hydroxamic acid
shRNA	short hairpin RNA
RNAi	RNA interference
VEGF	vascular endothelial growth factor
PDGF	platelet-derived growth factor
FGF	fibroblast growth factors
IGF-1	Insulin-like growth factor 1
TGF-β	transforming growth factor beta
IDO	indoleamine 2,3-dioxygenase
HGF	hepatocyte growth factor
EGF	epidermal growth factor
WNT	proto-oncogene protein
Cas	caspase
FADD	fas-associated protein with death domain
BID	BH3 interacting-domain death agonist
P53	tumor protein
Apaf-1	apoptotic protease activating factor 1
